# Distract Your Attention: Multi-Head Cross Attention Network for Facial Expression Recognition

**DOI:** 10.3390/biomimetics8020199

**Published:** 2023-05-11

**Authors:** Zhengyao Wen, Wenzhong Lin, Tao Wang, Ge Xu

**Affiliations:** 1 Fujian Provincial Key Laboratory of Information Processing and Intelligent Control, College of Computer and Control Engineering, Minjiang University, Fuzhou 350108, China; 2 School of Electrical and Mechanical Engineering, Fujian Agriculture and Forestry University, Fuzhou 350002, China; 3 The Key Laboratory of Cognitive Computing and Intelligent Information Processing of Fujian Education Institutions, Wuyi University, Wuyishan 354300, China; 4 Fujian Yilian-Health Nursing Information Technology Co., Ltd., Fuzhou 350003, China

**Keywords:** facial expression recognition, feature clustering network, multi-head attention network, attention fusion network

## Abstract

This paper presents a novel facial expression recognition network, called Distract your Attention Network (DAN). Our method is based on two key observations in biological visual perception. Firstly, multiple facial expression classes share inherently similar underlying facial appearance, and their differences could be subtle. Secondly, facial expressions simultaneously exhibit themselves through multiple facial regions, and for recognition, a holistic approach by encoding high-order interactions among local features is required. To address these issues, this work proposes DAN with three key components: Feature Clustering Network (FCN), Multi-head Attention Network (MAN), and Attention Fusion Network (AFN). Specifically, FCN extracts robust features by adopting a large-margin learning objective to maximize class separability. In addition, MAN instantiates a number of attention heads to simultaneously attend to multiple facial areas and build attention maps on these regions. Further, AFN distracts these attentions to multiple locations before fusing the feature maps to a comprehensive one. Extensive experiments on three public datasets (including AffectNet, RAF-DB, and SFEW 2.0) verified that the proposed method consistently achieves state-of-the-art facial expression recognition performance. The DAN code is publicly available.

## 1. Introduction

Facial expressions are direct and fundamental social signals in human communication [1,2]. Along with other gestures, they convey important nonverbal emotional cues in interpersonal relations. More importantly, vision-based facial expression recognition has become a powerful sentiment analysis tool in a wide spectrum of practical applications. For example, counseling psychologists assess a patient’s condition and consider treatment plans by constantly observing facial expressions [3]. In retail sales, a customer’s facial expression data are used to determine whether a human sales assistant is needed [4]. Other significant application areas include social robots, e-learning, and facial expression synthesis.

Facial expression recognition (FER) is a technology that uses computers to automatically recognize facial expressions. As this research area matures, a number of large-scale facial expression datasets have emerged. In an early seminal work [5], six prototypical emotional displays are postulated: angry (AN), disgust (DI), fear (FE), happy (HA), sad (SA), and surprise (SU), which are often referred to in the literature as *basic emotions*. Recent FER datasets regard neutral (NE) or contempt (CO) as additional expression categories, expanding the number of facial expression categories to seven or eight.

In contrast to generic image classification, there are strong common features among different categories of facial expressions. Indeed, multiple expressions share inherently similar underlying facial appearance, and their differences could be less distinguishable. In computer vision, a common strategy to address this issue involves adopting a variant of the center loss [6]. In this work, a Feature Clustering Network (FCN) is proposed, which includes a simple and straightforward extension to the center loss, and it works well for optimizing both intra-class and inter-class variations. Unlike existing methods [7,8,9], our method does not involve additional computations other than the variation of the cluster centers, and it only requires a few hyper-parameters.

**Figure 1:**
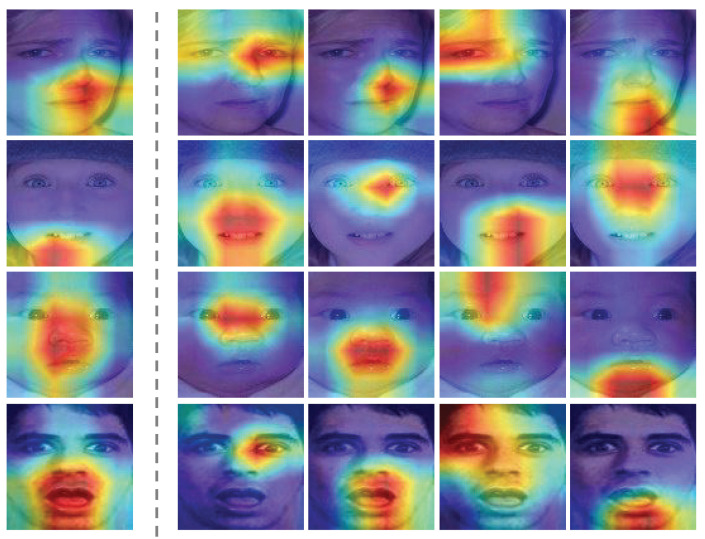
Comparing the Grad CAM++ visualization of a single attention head model and our proposed DAN on the RAF-DB test set. The first column is obtained with DACL [7], and the rest of the columns are generated by four attention heads from the proposed DAN model. Our method explicitly learns to attend to multiple local image regions for facial expression recognition.

In addition, one unique aspect of FER lies in the delicate contention between capturing the subtle local variations and obtaining a unified holistic representation. To attend to local details, some recent studies focus on attention mechanisms [7,10,11], achieving promising results. Nonetheless, as shown in Figure 1, it is difficult for a model with only a single attention head to concentrate on various parts of the face at the same time. In fact, it is our belief that facial expression is simultaneously manifested in multiple parts of the face, such as eyebrows, eyes, nose, mouth, chin, etc. Therefore, this paper proposes a Multi-head Attention Network (MAN) inspired by biological visual perception that instantiates a number of attention heads to attend to multiple facial areas. Our attention module implements both spatial and channel attentions, which allows for capturing higher-order interactions among local features while maintaining a manageable computational budget. Furthermore, this paper proposes an Attention Fusion Network (AFN) that ensures attention is drawn to multiple locations before their fusion into a comprehensive feature vector for downstream classification.

By integrating the above ideas, a novel facial expression recognition network, called Distract your Attention Network (DAN), is presented in this paper. Our method implements multiple attention heads and ensures that they capture useful aspects of the facial expressions without overlapping. Concretely, our work proposes three sub-networks, including a Feature Clustering Network (FCN), a Multi-head Attention Network (MAN), and an Attention Fusion Network (AFN). More specifically, our method first extracts and clusters the backbone feature embedding with FCN, where an affinity loss is applied to increase the inter-class distances while decreasing the intra-class distances. After that, an MAN is built to attend to multiple facial regions concurrently, where multiple attention heads that each include a spatial attention unit and a channel attention unit are adopted. Finally, the output feature vectors from MAN are fed to an AFN to output class scores. Specifically, this work designs a partition loss in AFN to force attention maps from the MAN to focus on different facial locations. As shown in Figure 1, a single attention module could only concentrate on one coarser image region, missing other important facial locations. On the contrary, our proposed DAN manages to simultaneously capture several vital facial regions.

The main contributions of our work are summarized as follows:

- To maximize class separability, this work proposes a simple yet effective feature clustering strategy in FCN to simultaneously optimize intra-class variations and inter-class margins.
- Our work demonstrates that a single attention module cannot sufficiently capture all the subtle and complex appearance variations across different expressions. To address this issue, MAN and AFN are proposed to capture multiple non-overlapping local attentions and fuse them to encode higher-order interactions among local features.
- The experimental results show that the proposed DAN method achieves an accuracy of 62.09% on AffectNet-8, 65.69% on AffectNet-7, 89.70% on RAF-DB, and 53.18% on SFEW 2.0, respectively, which represent the state of the art in facial expression recognition performance.

The rest of the paper is organized as follows. Section 2 reviews related literature in facial expression recognition with a particular focus on the attention mechanism and discriminative loss functions. Section 3 describes the proposed method in detail. Section 4 then presents the experimental evaluation results followed by closing remarks in Section 5.

## 2. Related Work

Facial expression recognition is an image classification task involving accurately identifying the emotional state of humans. The earliest study on FER dates back to 1872, when Darwin first proposed the argument of consistency in expressions [2]. In 1971, Ekman and Friesen presented the six basic emotions [5]. Later, the first facial expression recognition system [12] was proposed in 1991, which was based on optical flow. After that, the FER system has gradually matured and, in general, can be divided into three sub-processes: face detection, feature extraction, and expression classification [13]. Recently, FER systems are benefiting from the rapid development of deep learning, and a unified neural network can be used to perform both feature extraction and expression classification.

One of the most significant applications of FER is in human–computer interaction. By analyzing users’ facial expressions, computers can interpret human emotions and respond accordingly, providing more natural and intuitive user interfaces. This technology has already been implemented in various areas, such as gaming, virtual reality, and video conferencing. In the healthcare industry, facial expression recognition can be used to detect and diagnose various mental health disorders. It can also be used to monitor patients’ emotions and provide personalized treatment options. For example, Ref. [14] take advantage of FER instead of medical devices to detect a people’s health state. FER also has important applications in artistic research, Ref. [15] use FER to recognize facial expressions in opera performance scenes to help teams assess user satisfaction and use it to make adjustments. Very recently, Ref. [16] propose a method for synthesizing recognizable face line portraits with controllable expression and high recognizability based on a triangle coordinate system.

**Attention mechanism.** Attention mechanism plays an important role in visual perception [17,18]. In particular, attention enables human beings to actively seek more valuable information in a complex scene. In recent years, there have been a plethora of studies attempting to introduce attention mechanisms into deep Convolutional Neural Network (CNN) with success. For example, Ref. [19] focus on the channel relationship of network features and propose a squeeze-and-excitation block to retain the most valuable channel information. Ref. [20] introduce frequency analysis to attention mechanism and present a new method termed FcaNet to compress information loss in scalar-based channel representations. Ref. [21] present a group-wise spatial attention module (SGE) where the spatial-wise features are divided into multiple groups to learn a potential spatial connection. By leveraging the complementary nature between channel-wise and spatial-wise features, Ref. [22] propose the Convolutional Block Attention Module (CBAM) that sequentially connects a channel attention and spatial attention to obtain rich attention features. Ref. [23] propose a coordinate attention that embeds positional information into channel attention to generate spatially selective attention maps. Ref. [24] propose a new triplet attention that captures cross-dimensional interaction to efficiently build inter-dimensional dependencies. Likewise, Ref. [25] use a position attention module and a channel attention module in parallel to share the local-wise and global-wise features for scene segmentation task. Very recently, seminal works based on self-attention mechanisms have emerged, such as Swin-Transformer [26], DAB-DETR [27], DaViT [28], and QFormer [29]. These models have indeed demonstrated superior performance, but they also bring with them a huge number of model parameters and an expensive computational cost, which are unsuitable for lightweight applications. Inspired by these efforts, our method includes design of an attention head that sequentially cascades a spatial attention and a channel attention unit, which is both efficient and effective.

There are a few papers that introduce the above progress into FER. For example, Ref. [30] apply region attention on the CNN backbone to enhance its power of capturing local information. Ref. [31] construct a bottom-up and top-down architecture to obtain low resolution attention features. In these papers, only a single attention head is used, which would generally lead to attention on a rough area of the face. In our work, however, multiple non-overlapping attention regions could be simultaneously activated to capture information from different local regions. This is particularly useful for FER, as we need to simultaneously attend to multiple regions (e.g., eyes, nose, mouth, forehead, and chin) to capture the subtle differences among emotion classes.

Attention mechanisms have found applications in a wide range of areas in computer vision. For example, Ref. [32] propose a joint weak saliency and attention-aware model for person re-identification, in which saliency features are weakened to obtain more complete global features and diversified saliency features via attention diversity. Ref. [33] propose a reconstruction method based on attention mechanism to address the issue of low-frequency and high-frequency components being treated equally in image super-resolution. Ref. [34] propose a multi-head self-attention approach for multi-label mRNA subcellular localization prediction and achieve excellent results.

**Discriminative loss.** A discriminative loss function can strongly regulate the distribution of deep features. For example, Ref. [35] propose contrastive loss, which is an efficient loss function that maximizes class separability. In detail, a general Euclidean metric is used for features from the same class, but for diverse classes, the loss values will get close to a maximum margin. In addition, Ref. [6] present the center loss to learn a center distribution of each class and penalize the distances between deep features and their corresponding class centers. Differently, Ref. [36] propose using an angle as a distance measure and introduce an angular softmax loss. That work is followed by a number of methods [37,38,39] that improve the angular loss function.

In recent years, several studies demonstrate that discriminative loss functions could be well adapted to the FER task. Ref. [40] combine the advantages of center loss and softmax loss and propose a discriminative distribution-agnostic loss function. Concretely, a center loss aggregates the features of the same class into a cluster, and a softmax loss separates the adjacent classes. Similarly, Ref. [8] introduce a cosine metric based on center loss to increase the inter-class distance among different categories. Furthermore, Ref. [7] propose an attentive center loss, which advocates learning the relationship of each class center for the center loss. However, all these loss functions bring in auxiliary parameters and computations. On the contrary, the affinity loss proposed in this paper is more simple and uses the internal relationship among class centers to increase the inter-class distance.

## 3. Our Approach

This section describes the proposed DAN model in detail. In order to learn high-quality attentive features, our DAN is divided into three components: Feature Clustering Network (FCN), Multi-head Attention Network (MAN), and Attention Fusion Network (AFN). Firstly, the FCN accepts a batch of face images and outputs basic feature embedding with class discrimination abilities. Afterwards, the MAN is employed to learn diverse attention maps that capture several sectional facial expression regions. Then, these attention maps are explicitly trained to focus on different areas by the AFN. Finally, the AFN fuses features coming from all attention heads and gives a prediction for the expression category of the input images.

**Figure 2:**
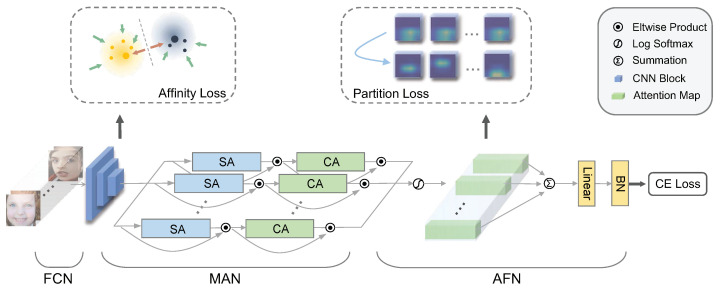
Overview of our proposed DAN. The method is composed of three sub-networks. The backbone features are first extracted and clustered by a Feature Clustering Network (FCN), where an affinity loss is applied to increase the inter-class margin and to reduce the intra-class variance. Next, a Multi-head Attention Network (MAN) is built to attend to multiple facial regions concurrently by a series of Spatial Attention (SA) and Channel Attention (CA) units. Finally, an Attention Fusion Network (AFN) regulates the attention maps by enforcing variance among the attention feature vectors and outputs a class confidence.

In particular, the presented MAN contains a series of lightweight but effective attention heads. An attention head composes of a spatial attention unit and a channel attention unit in sequential order. Distinctively, the spatial attention unit involves convolution kernels of various sizes. A channel attention unit is connected to the end of the spatial attention unit to reinforce the attention maps by simulating an encoder–decoder structure. Both the spatial attention and the channel attention units are integrated back into the input features. The overall process of the proposed DAN is shown in Figure 2.

### 3.1. Feature Clustering Network (FCN)

Let us begin by introducing the FCN. Considering both performance and the number of parameters in our model, our method employs a residual network [41] as the backbone. As discussed earlier, different facial expressions may share similar underlying facial appearance. Therefore, this paper proposes a discriminative loss function, named affinity loss, to maximize class margins. Concretely, in each training step, our method encourages features to move closer to the class center to which they belong. At the same time, our method pushes centers of different classes apart in order to maintain good separability.

More formally, suppose we have the *i*-th input image feature $x_{i}\in X$ with a class label $y_{i}\in Y$ during training, where X is the input feature space and Y is the label space. Here, i∈{1⋯M} where *M* is the number of images in the training set. For the sake of simplicity, the output features of our backbone can be written as:

(1) $$x_{i}^{{}^{\prime }}=F_{r}\left(w_{r},x_{i}\right)$$

where $F_{r}$ represents the backbone network and $w_{r}$ denotes the network parameters of $F_{r}$.

**Affinity loss.** The affinity loss is proposed to maximize the inter-class distance while minimizing the intra-class variation. By virtue of the affinity loss, the backbone network can accurately cluster various features of facial expressions toward their respective class centers. Compared to the standard center loss [6], our affinity loss advocates that it makes sense to expand the distance between class centers, because a larger distance between class centers further prevents overlap among classes. Formally, given the class center matrix $c\in R^{D\times |Y|}$ wherein each column corresponds to the center of a specific class, the affinity loss function can be written as follows:

(2) $$L_{af}=\frac{\sum_{i=1}^{N}\left|\right|x_{i}^{{}^{\prime }}-c_{y_{i}}{\left|\right|}_{2}^{2}}{\sigma_{c}^{2}}$$

where *N* is the number of images in a training batch, *D* is the dimension of class centers, $c_{y_{i}}$ is the column vector in *c* that corresponds to the ground-truth class of the *i*-th image, and $\sigma_{c}$ indicates the standard deviation among class centers. One might notice that $x_{i}^{{}^{\prime }}$ is not necessarily a feature vector and could instead be a feature map of higher dimensions. In practice, following [6], a global average pooling is performed on $x_{i}^{{}^{\prime }}$, and any singleton dimensions are then removed before applying Equation (2), and we note that similar operations to flatten the feature maps may also work.

**Figure 3:**
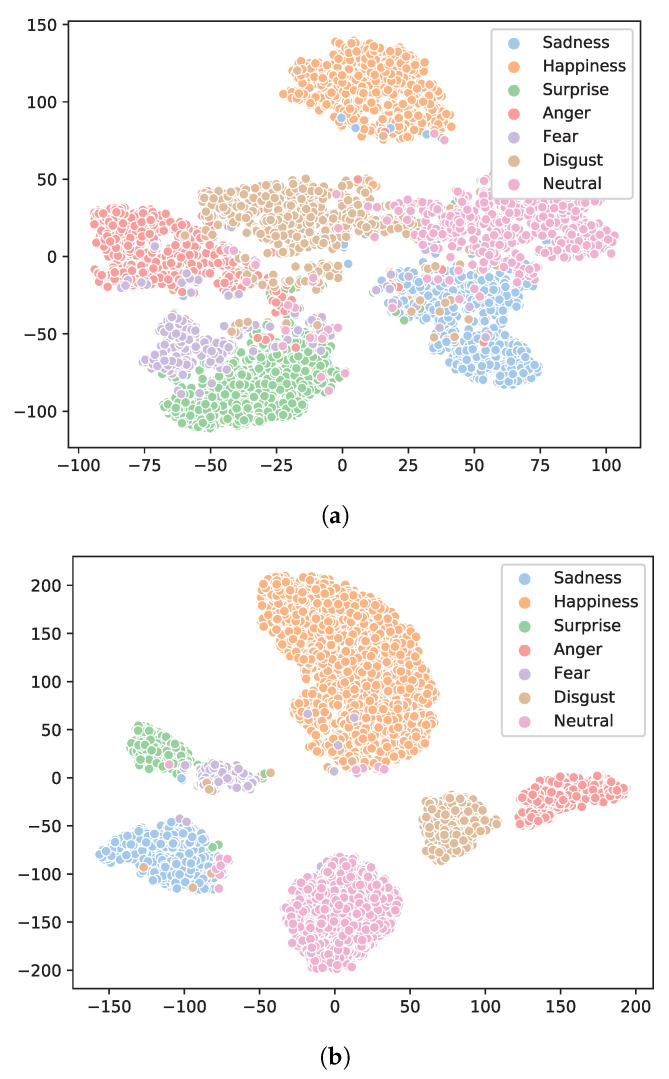
The t-SNE visualization of features obtained with standard center loss (**a**) and the proposed affinity loss (**b**) on the RAF-DB dataset with our FCN, color-coded according to their class membership. It is clear that our affinity loss provides much better class separability by optimizing both the inter-class margins and the intra-class variations.

Despite its simplicity, our affinity loss is more effective than the standard center loss, as it encourages wider margins among classes as a result of pushing class centers further away from each other. This arguably completes the other half of the puzzle missing in the center loss, as the standard center loss only enforces tightness within classes but not sparsity among different classes. In particular, $\sigma_{c}$ can be readily available during training, as class centers have already been computed, and no additional hyper-parameters are needed for our affinity loss. Figure 3 presents the t-SNE visualization of the features obtained by FCN with center loss and affinity loss, respectively. It is clear that by integrating the affinity loss, our FCN learns feature clusters of better quality, and the class margins are clearly wider among different classes.

### 3.2. Multi-Head Attention Network (MAN)

**Figure 4:**
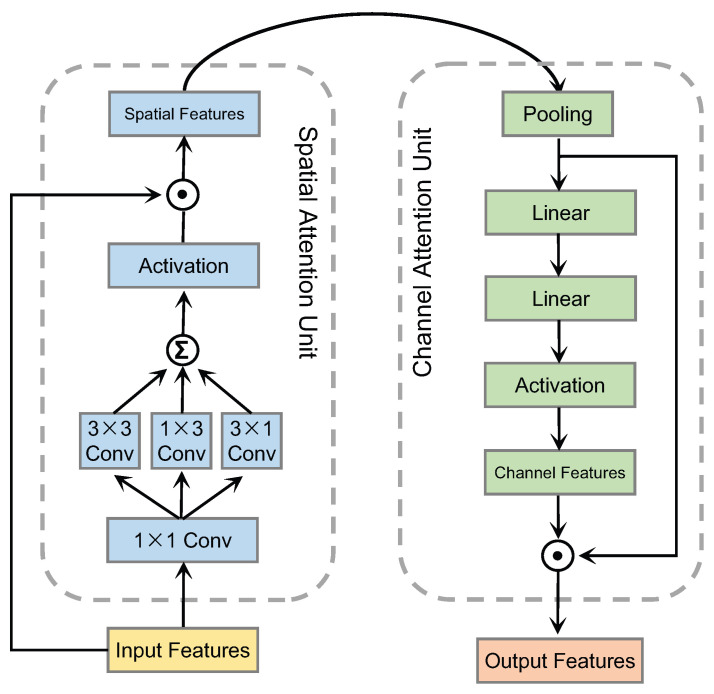
The structure of the proposed attention head, which consists of a spatial attention unit and a channel attention unit. The spatial attention unit and the channel attention unit work together to enable the attention head to selectively focus on relevant spatial locations and channel features.

As discussed earlier, a single attention head may be insufficient for effective FER, and the joint reasoning of multiple local regions is necessary. In this regard, our MAN contains a few parallel heads, which remain independent of each other. As shown in Figure 4, an attention head is a combination of a spatial attention unit and a channel attention unit. The spatial attention unit receives the input features from the FCN and extracts the spatial features. Then, the channel attention unit accepts the spatial features as the input feature and extracts the channel features. Features from the two above dimensions are then combined into an attention vector. In particular, it should be noted that all parallel heads are identical in terms of their sizes, differing only in parameter values.

More concretely, the left section of Figure 4 illustrates the spatial attention unit, which consists of four convolutional layers and one activation function. In particular, our method constructs the 1×1,1×3,3×1, and 3×3 convolution kernels to capture local features at multiple scales. The channel attention unit shown in the right part consists of a global average pooling layer, two linear layers, and one activation function. In particular, our method takes advantage of having two linear layers to encode the channel information.

Formally, let $H=\{H_{1},\ldots ,H_{K}\}$ be the spatial attention heads and $S=\{s_{1},\ldots ,s_{K}\}$ be the output spatial attention maps, where *K* is the number of attention heads. Given the output features of our backbone $x^{{}^{\prime }}$ (the subscript *i* is omitted for notation brevity), the output of the *j*-th spatial attention unit can then be written as:

(3) $$s_{j}=x^{{}^{\prime }}⊙H_{j}\left(w_{s},x^{{}^{\prime }}\right),j\in \left\{1,\ldots ,K\right\}$$

where $w_{s}$ are the network parameters of $H_{j}$, and ⊙ is the Hadamard product.

Similarly, assume $H^{{}^{\prime }}=\left\{H_{1}^{{}^{\prime }},\ldots ,H_{K}^{{}^{\prime }}\right\}$ are the channel attention heads and $A=\{a_{1},\ldots ,a_{K}\}$ are the final attention feature vectors of MAN. The *j*-th output $a_{j}$ can be written as:

(4) $$a_{j}=s_{j}⊙H_{j}^{{}^{\prime }}\left(w_{c},s_{j}\right),j\in \left\{1,\ldots ,K\right\}$$

where $w_{c}$ are the network parameters of $H_{j}^{{}^{\prime }}$.

The key benefits of the proposed MAN are two-fold. Firstly, our method incorporates spatial attention and channel attention sequentially for each attention head, so as to efficiently capture high-order interactions among features. More importantly, our method instantiates multiple attention heads so that the model can attend to different facial regions at the same time, which is not possible with a single attention head.

### 3.3. Attention Fusion Network (AFN)

Furthermore, to guide the MAN in learning attention maps in an orchestrated fashion, this work proposes the AFN to further enhance the features learned by MAN. Firstly, AFN scales the attention feature vectors by applying a log-softmax function to emphasize the most interesting region. After that, a partition loss is proposed to instruct the attention heads to focus on different crucial regions and avoid overlapping attentions. This would ensure the efficacy of multiple attention heads, making them attend to non-overlapping local regions. Lastly, the normalized attention feature vectors are merged into one, and we can then compute class confidence with a linear layer.

**Feature scaling.** The final attention feature vectors A are firstly scaled using a log-softmax function along the dimension corresponding to the attention heads, so as to obtain a common metric for the features from different heads. More specifically, the output of MAN from Section 3.2 gives the attention feature vectors $A=\{a_{1},\ldots ,a_{K}\}$, and we assume that any of these vectors, e.g., the *j*-th vector $a_{j}$, is *L*-dimensional (e.g., L=512 in our experiments). In this case, the feature scaling result $v_{j}$ for $a_{j}$ can be given by:

(5) $$v_{j}^{l}=\log\left(\frac{\exp(a_{j}^{l})}{\sum_{k=1}^{K}\exp\left(a_{k}^{l}\right)}\right),l\in \left\{1,\ldots ,L\right\},j\in \left\{1,\ldots ,K\right\}$$

where $a_{j}^{l}$ is the *l*-th element of $a_{j}$, and $v_{j}^{l}$ is the *l*-th element of $v_{j}$. After scaling, the features from different heads are further added up for the final classification, as shown in Figure 2.

**Partition loss.** This paper proposes a partition loss to direct the attention heads to focus on different facial feature regions so they will not collapse into a single attention. As visualized in the right part of Figure 1, our partition loss successfully controls the heads of MAN to follow different facial areas without additional interventions. More specifically, we do so by maximizing the variance among attention vectors. In particular, our method considers *K*, the number of attention heads, as a parameter to adaptively adjust the descent speed of loss values. The MAN with a larger quantity of attention heads may generate more subtle areas of attention. Overall, we write our partition loss as follows:

(6) $$L_{pt}=\frac{1}{NL}\sum_{i=1}^{N}\sum_{l=1}^{L}\log\left(1+\frac{K}{\sigma_{i,l}^{2}}\right)$$

where *N* is the number of images in the current batch, *L* is the dimension of the attention feature vector in each head, $\sigma_{i,l}^{2}$ denotes the variance of $v_{j}^{l}$ where j∈{1,…,K} when the *i*-th image and the *l*-th dimension are given.

### 3.4. Model Learning

As shown above, our DAN model is comprised of three components: FCN, MAN, and AFN. In order to train this model, one has to consider the losses from all components (i.e., the affinity loss from FCN, the partition loss from AFN, and the cross-entropy loss for image classification) within a unified framework. Following the standard practice in deep learning, our method learns a final loss function by integrating all these loss functions:

(7) $$L=\lambda_{1}L_{af}+\lambda_{2}L_{pt}+L_{cls}$$

where $\lambda_{1}$, $\lambda_{2}$ are the weighting hyper-parameters for $L_{af}$ and $L_{pt}$. $L_{cls}$ denotes the cross-entropy loss in image classification. In our experiments, we empirically set both $\lambda_{1}$ and $\lambda_{2}$ to 1.0 and note that the consistent performance gains we observe are not particularly sensitive to their values.

## 4. Experimental Evaluation

This section describes our experimental evaluation results in detail. In particular, the superiority of the proposed method is quantitatively verified on three benchmark datasets: AffectNet, RAF-DB, and SFEW 2.0. This paper shows that the proposed method provides consistent performance improvements to a number of strong baselines. In addition, it is demonstrated that the various components of our model are all contributing to the final performance through ablation studies.

### 4.1. Datasets

#### 4.1.1. AffectNet

AffectNet [42] is a large-scale database of facial expressions that contains two benchmark branches: AffectNet-7 and AffectNet-8 (with an additional category of “contempt”). AffectNet-7 includes 287,401 images with seven classes, where all images are divided into 283,901 training samples and 3500 testing samples. Additionally, AffectNet-8 introduces the contempt data and expands the number of training and test samples to 287,568 and 4000, respectively.

#### 4.1.2. RAF-DB

RAF-DB [43] is a real-world database with more than 29,670 facial images downloaded from the Internet. Seven basic and eleven compound emotion labels are provided for the dataset through manual labeling. There are 15,339 images in total for expression classification (including 12,271 training set and 3068 testing set images), each of which is aligned and cropped to a size of 100×100.

#### 4.1.3. SFEW 2.0

SFEW 2.0 [44] is the newest version for SFEW dataset in which each facial emotion is extracted from the static frames of the AFEW video database. It is divided into three sets of seven expression categories: train (958 samples), validate (436 samples), and test (372 samples). Compared to the size of AffectNet and RAF-DB, SFEW 2.0 is light and compact.

### 4.2. Implementation Details

On RAF-DB and AffectNet datasets, our work directly uses the official aligned image samples. For the SFEW 2.0 dataset, the facial images are manually aligned using the RetinaFace [45] model. Input images are resized to 224×224 pixels for each training and testing step on all datasets. A few basic random data augmentation methods (such as horizontal flipping, random rotation, and erasing) are used selectively to prevent over-fitting. Moreover, a ResNet-18 [41] model is adopted as the backbone of FCN in all experiments, and for a fair comparison, this work pre-trains the ResNet-18 model on the MS-Celeb-1M face recognition dataset.

Our experimental code is implemented with PyTorch, and the models are trained on a workstation with an NVIDIA Tesla P100 12GB GPU. Our code is publicly available from https://github.com/yaoing/DAN. For all tasks, models are trained for 40 epochs with a uniform batch size of 256 (mainly due to GPU memory constraint), and the head number of attention in MAN is set to 4 by default.

More specifically, this work trains our model on the RAF-DB dataset using the SGD algorithm with an initial learning rate of 0.1. For the AffectNet-7 and AffectNet-8 datasets, the models are optimized by the ADAM algorithm with a smaller learning rate of 0.0001. Moreover, considering the inconsistent ratio of training set to testing set, this work introduces a dataset sampling strategy for the training step, i.e., upsampling the low-volume categories and downsampling the high-volume categories to obtain a more balanced dataset.

### 4.3. Quantitative Performance Comparisons

This paper presents the quantitative performance comparison results in Table 1, Table 2, Table 3 and Table 4 for AffectNet, RAF-DB, and SFEW 2.0. Our proposed method achieves an accuracy of 62.09% on AffectNet-8 and an accuracy of 65.69% on AffectNet-7, which are both superior to existing methods. Comparing for the RAF-DB dataset, DAN acquires 89.70% in accuracy, which is state of the art. Our method, although not the best, also obtains a competitive accuracy of 53.18% on the more compact SFEW 2.0 dataset. This is perhaps because the multi-head attention would require larger datasets for learning to ensure an effective model. Overall, the results above clearly demonstrate that the proposed method is highly competitive and the components in our method are effective across multiple datasets.

### 4.4. Ablation Studies and Computational Complexity

#### 4.4.1. Effects of Loss Functions for FCN and AFN

In order to verify the efficacy of the individual loss functions proposed in our method, this paper now performs ablation studies on the RAF-DB dataset to demonstrate that our affinity loss and partition loss are indeed contributing to model performance. Specifically, this work evaluates the influence of using different loss functions for FCN and AFN separately in Table 5. In FCN, our proposed affinity loss provides a considerable performance improvement to the standard center loss. Similarly, the partition loss plays a crucial role in the performance of AFN. These results demonstrate that the affinity loss and the partition loss both contribute to the superior performance of our model.

**Table 1:** Performance comparison on the AffectNet-8 dataset.

Methods	Accuracy (%)
PhaNet [46]	54.82
ESR-9 [47]	59.30
RAN [48]	59.50
SCN [49]	60.23
PSR [50]	60.68
EfficientFace [51]	59.89
EfficientNet-B0 [52]	61.32
MViT [53]	61.40
ResNet-18	56.84
DAN (ours)	**62.09**

**Table 2:** Performance comparison on the AffectNet-7 dataset.

Methods	Accuracy (%)
Separate-Loss [54]	58.89
FMPN [55]	61.25
LDL-ALSG [56]	59.35
VGG-FACE [57]	60.00
OADN [58]	61.89
DDA-Loss [40]	62.34
EfficientFace [51]	63.70
MViT [53]	64.57
ResNet-18	56.97
DAN (ours)	**65.69**

**Table 3:** Performance comparison on the RAF-DB dataset.

Methods	Accuracy (%)
Separate-Loss [54]	86.38
DDA-Loss [40]	86.9
SCN [49]	87.03
PSR [50]	88.98
DACL [7]	87.78
IF-GAN [59]	88.33
EfficientFace [51]	88.36
MViT [53]	88.62
ResNet-18	86.25
DAN (ours)	**89.70**

**Table 4:** Performance comparison on the SFEW 2.0 dataset.

Methods	Accuracy (%)
IACNN [60]	50.98
DLP-CNN [43]	51.05
Island Loss [8]	52.52
TDTLN [61]	53.10
RAN [48]	54.19
LDL-ALSG [56]	56.50
ViT + SE [62]	54.29
FaceCaps [63]	**58.50**
Baseline (ResNet-18)	51.29
DAN (ours)	53.18

**Table 5:** Ablation studies for the loss functions in FCN and AFN on the RAF-DB dataset. The proposed affinity loss and partition loss both provide superior performance. Note that the cross-entropy loss is used in all cases.

Task	Methods	Accuracy (%)
FCN	-	88.17
	center loss	88.91
	affinity loss	89.70
AFN	-	88.20
	partition loss	89.70

#### 4.4.2. Number of Attention Heads

**Figure 5:**
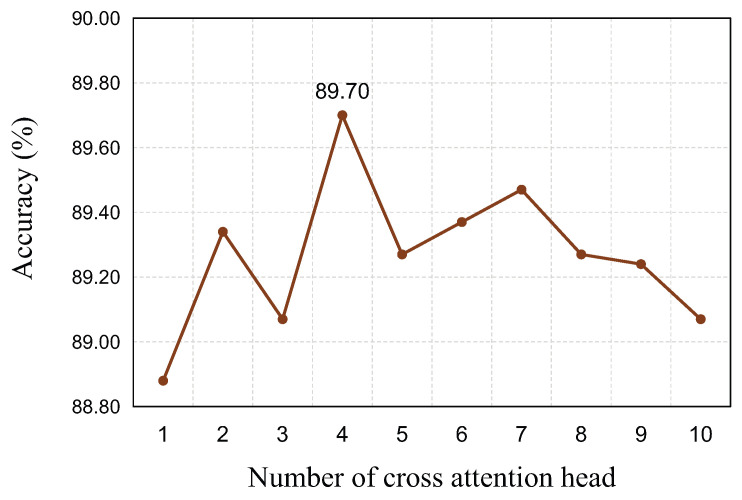
Ablation studies for the changing number of attention heads of MAN on the RAF-DB dataset. It is clear that our empirically chosen model with four heads is superior to the model with a single attention head.

In addition, the number of attention heads obviously affects the performance of our model. Figure 5 shows the accuracy results with a changing number of attention heads on the RAF-DB dataset. It is evident that our proposed MAN structures are superior to a single attention module. Moreover, using four attention heads maximizes the performance gain. Therefore, four attention heads are used throughout the experiments in this paper.

#### 4.4.3. Computational Complexity

Table 6 compares our method against state-of-the-art methods in terms of model size and inference time. Our DAN with four attention heads uses 19.72 M and 2.23 G of parameters and FLOPs, respectively, which corresponds to moderate resource consumption, to achieve its state-of-the-art facial expression recognition performance.

### 4.5. Confusion Matrix

**Figure 6:**
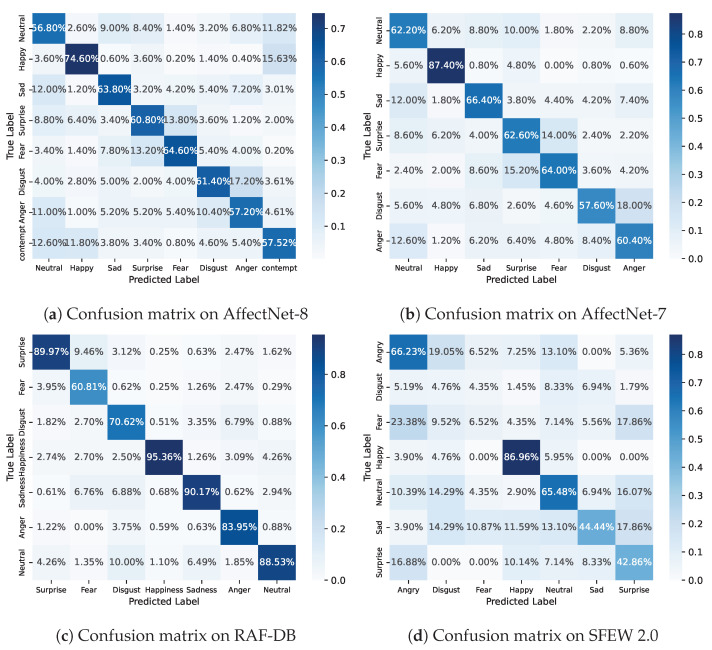
Confusion matrix on AffectNet-8, AffectNet-7, RAF-DB, and SFEW 2.0, respectively.

In order to better understand the performance of our model on each facial expression category, Figure 6 presents the confusion matrices on each dataset we evaluated. More specifically, it is obvious that the “Happy” class is the easiest on both Affect-8 and Affect-7, followed by “Sad”, “Surprise”, and “Fear”. On RAF-DB, our DAN model has a high accuracy in “Happiness”, “Sadness”, “Surprise”, “Neutral”, and “Anger” classes. On SFEW 2.0, our method performs relatively well on the “Happy” and “Neutral” classes, while the “Disgust” and “Fear” classes are very challenging. Possible reasons for the large gaps in the performance include the appearance similarities among facial expression categories as well as the skewed class distribution in the training dataset. It is therefore obvious that to further improve the performance of our method in the future, extra efforts should be made to avoid class confusion on the more difficult classes such as “Disgust”, “Fear”, and “Anger”.

### 4.6. Precision–Recall Analysis

**Figure 7:**
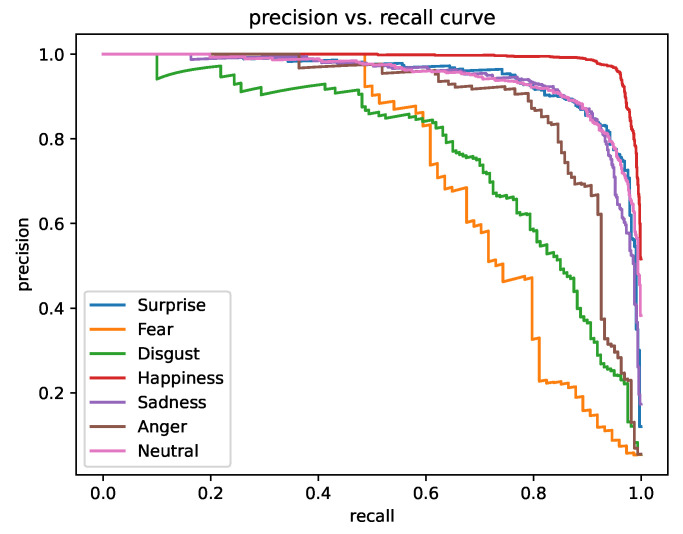
The per-class precision–recall curves on RAF-DB. Our method provides near-perfect precision for most classes in the low recall regime. Classes such as “Happiness”, “Neutral”, “Surprise”, and “Sadness” are relatively easy, while the disgust and fear classes are the most challenging.

Finally, Figure 7 presents the per-class precision–recall curves on the RAF-DB dataset. It is evident that our approach achieves near-perfect performance in “Happiness” class, and also performs well in “Neutral”, “Surprise”, “Sadness”, and “Anger” classes. Overall, our method provides over 80% precision at 50% recall for all classes.

## 5. Conclusions

This paper presents a robust method for facial expression recognition that consists of three novel sub-networks including the Feature Clustering Network (FCN), the Multi-head Attention Network (MAN), and the Attention Fusion Network (AFN). Specifically, the FCN learns to maximize class separability for backbone facial expression features, the MAN captures multiple diverse attentions, and the AFN penalizes overlapping attentions and fuses the learned features. Experimental results on three benchmark datasets demonstrate the superiority of our method for FER. It is our hope that the exploration into feature clustering and learning multiple diverse attentions would provide insights for future research in facial expression recognition and other related vision tasks.

## Figures and Tables

**Table 6:** Comparison based on model size and inference time. Our method provides competitive performance while maintaining a manageable computational cost.

Methods	Params (M)	FLOPs (G)
EfficientFace [51]	1.28	0.15
SCN [49]	11.18	1.82
RAN [48]	11.19	14.55
PSR[50]	20.24	10.12
DACL [7]	103.04	1.91
ResNet 18	11.69	1.82
DAN (ours)	19.72	2.23

## Data Availability

The data presented in this study are openly available in https://github.com/yaoing/DAN.

## References

[1] Ekman P., Rosenberg E.L. (1997). What the Face Reveals: Basic and Applied Studies of Spontaneous Expression Using the Facial Action Coding System (FACS).

[2] Darwin C. (2015). The Expression of the Emotions in Man and Animals.

[3] Fasel B., Luettin J. (2003). Automatic facial expression analysis: A survey. Pattern Recognit..

[4] Shergill G.S., Sarrafzadeh A., Diegel O., Shekar A. (2008). Computerized Sales Assistants: The Application of Computer Technology to Measure Consumer Interest-A Conceptual Framework.

[5] Ekman P., Friesen W.V. (1971). Constants across cultures in the face and emotion. J. Personal. Soc. Psychol..

[6] Wen Y., Zhang K., Li Z., Qiao Y. (2016). A discriminative feature learning approach for deep face recognition. Proceedings of the European Conference on Computer Vision.

[7] Farzaneh A.H., Qi X. Facial expression recognition in the wild via deep attentive center loss. Proceedings of the IEEE/CVF Winter Conference on Applications of Computer Vision.

[8] Cai J., Meng Z., Khan A.S., Li Z., O’Reilly J., Tong Y. Island loss for learning discriminative features in facial expression recognition. Proceedings of the 2018 13th IEEE International Conference on Automatic Face & Gesture Recognition (FG 2018).

[9] Li Z., Wu S., Xiao G. Facial expression recognition by multi-scale cnn with regularized center loss. Proceedings of the 2018 24th International Conference on Pattern Recognition (ICPR).

[10] Fernandez P.D.M., Pena F.A.G., Ren T.I., Cunha A. (2019). Feratt: Facial expression recognition with attention net. arXiv.

[11] Li J., Jin K., Zhou D., Kubota N., Ju Z. (2020). Attention mechanism-based CNN for facial expression recognition. Neurocomputing.

[12] Mase K. (1991). Recognition of facial expression from optical flow. IEICE Trans. Inf. Syst..

[13] Wu T., Fu S., Yang G. (2012). Survey of the facial expression recognition research. Proceedings of the International Conference on Brain Inspired Cognitive Systems.

[14] Bibbo’ L., Cotroneo F., Vellasco M. (2023). Emotional Health Detection in HAR: New Approach Using Ensemble SNN. Appl. Sci..

[15] Ceccacci S., Generosi A., Giraldi L., Mengoni M. (2023). Emotional Valence from Facial Expression as an Experience Audit Tool: An Empirical Study in the Context of Opera Performance. Sensors.

[16] Dong X., Ning X., Xu J., Yu L., Li W., Zhang L. (2023). A Recognizable Expression Line Portrait Synthesis Method in Portrait Rendering Robot. IEEE Trans. Comput. Soc. Syst..

[17] Rensink R.A. (2000). The dynamic representation of scenes. Vis. Cogn..

[18] Corbetta M., Shulman G.L. (2002). Control of goal-directed and stimulus-driven attention in the brain. Nat. Rev. Neurosci..

[19] Hu J., Shen L., Sun G. Squeeze-and-excitation networks. Proceedings of the IEEE Conference on Computer Vision and Pattern Recognition, Salt Lake City.

[20] Qin Z., Zhang P., Wu F., Li X. Fcanet: Frequency channel attention networks. Proceedings of the IEEE/CVF International Conference on Computer Vision.

[21] Li X., Hu X., Yang J. (2019). Spatial group-wise enhance: Improving semantic feature learning in convolutional networks. arXiv.

[22] Woo S., Park J., Lee J.Y., Kweon I.S. Cbam: Convolutional block attention module. Proceedings of the European Conference on Computer Vision (ECCV).

[23] Hou Q., Zhou D., Feng J. Coordinate attention for efficient mobile network design. Proceedings of the IEEE/CVF Conference on Computer Vision and Pattern Recognition.

[24] Misra D., Nalamada T., Arasanipalai A.U., Hou Q. Rotate to attend: Convolutional triplet attention module. Proceedings of the IEEE/CVF Winter Conference on Applications of Computer Vision.

[25] Fu J., Liu J., Tian H., Li Y., Bao Y., Fang Z., Lu H. Dual attention network for scene segmentation. Proceedings of the IEEE/CVF Conference on Computer Vision and Pattern Recognition.

[26] Liu Z., Lin Y., Cao Y., Hu H., Wei Y., Zhang Z., Lin S., Guo B. (2021). Swin transformer: Hierarchical vision transformer using shifted windows. arXiv.

[27] Liu S., Li F., Zhang H., Yang X., Qi X., Su H., Zhu J., Zhang L. (2022). Dab-detr: Dynamic anchor boxes are better queries for detr. arXiv.

[28] Ding M., Xiao B., Codella N., Luo P., Wang J., Yuan L. (2022). Davit: Dual attention vision transformers. Proceedings of the Computer Vision–ECCV 2022: 17th European Conference.

[29] Zhang Q., Zhang J., Xu Y., Tao D. (2023). Vision Transformer with Quadrangle Attention. arXiv.

[30] Xie S., Hu H., Wu Y. (2019). Deep multi-path convolutional neural network joint with salient region attention for facial expression recognition. Pattern Recognit..

[31] Zhu K., Du Z., Li W., Huang D., Wang Y., Chen L. Discriminative attention-based convolutional neural network for 3D facial expression recognition. Proceedings of the 2019 14th IEEE International Conference on Automatic Face & Gesture Recognition (FG 2019).

[32] Ning X., Gong K., Li W., Zhang L. (2021). JWSAA: Joint weak saliency and attention aware for person re-identification. Neurocomputing.

[33] Chen Y., Liu L., Phonevilay V., Gu K., Xia R., Xie J., Zhang Q., Yang K. (2021). Image super-resolution reconstruction based on feature map attention mechanism. Appl. Intell..

[34] Wang D., Zhang Z., Jiang Y., Mao Z., Wang D., Lin H., Xu D. (2021). DM3Loc: Multi-label mRNA subcellular localization prediction and analysis based on multi-head self-attention mechanism. Nucleic Acids Res..

[35] Hadsell R., Chopra S., LeCun Y. Dimensionality reduction by learning an invariant mapping. Proceedings of the 2006 IEEE Computer Society Conference on Computer Vision and Pattern Recognition (CVPR’06).

[36] Liu W., Wen Y., Yu Z., Li M., Raj B., Song L. Sphereface: Deep hypersphere embedding for face recognition. Proceedings of the IEEE Conference on Computer Vision and Pattern Recognition.

[37] Liu Y., Li H., Wang X. (2017). Learning deep features via congenerous cosine loss for person recognition. arXiv.

[38] Wang H., Wang Y., Zhou Z., Ji X., Gong D., Zhou J., Li Z., Liu W. Cosface: Large margin cosine loss for deep face recognition. Proceedings of the IEEE Conference on Computer Vision and Pattern Recognition, Salt Lake City.

[39] Deng J., Guo J., Xue N., Zafeiriou S. Arcface: Additive angular margin loss for deep face recognition. Proceedings of the IEEE/CVF Conference on Computer Vision and Pattern Recognition.

[40] Farzaneh A.H., Qi X. Discriminant distribution-agnostic loss for facial expression recognition in the wild. Proceedings of the IEEE/CVF Conference on Computer Vision and Pattern Recognition Workshops.

[41] He K., Zhang X., Ren S., Sun J. Deep residual learning for image recognition. Proceedings of the IEEE Conference on Computer Vision and Pattern Recognition.

[42] Dhall A., Goecke R., Lucey S., Gedeon T. (2012). Collecting large, richly annotated facial-expression databases from movies. IEEE Multimed..

[43] Li S., Deng W. (2018). Reliable crowdsourcing and deep locality-preserving learning for unconstrained facial expression recognition. IEEE Trans. Image Process..

[44] Dhall A., Goecke R., Lucey S., Gedeon T. Static facial expression analysis in tough conditions: Data, evaluation protocol and benchmark. Proceedings of the 2011 IEEE International Conference on Computer Vision Workshops (ICCV Workshops).

[45] Deng J., Guo J., Zhou Y., Yu J., Kotsia I., Zafeiriou S. (2019). Retinaface: Single-stage dense face localisation in the wild. arXiv.

[46] Liu Y., Peng J., Zeng J., Shan S. (2019). Pose-adaptive hierarchical attention network for facial expression recognition. arXiv.

[47] Siqueira H., Magg S., Wermter S. Efficient facial feature learning with wide ensemble-based convolutional neural networks. Proceedings of the AAAI Conference on Artificial Intelligence.

[48] Wang K., Peng X., Yang J., Meng D., Qiao Y. (2020). Region attention networks for pose and occlusion robust facial expression recognition. IEEE Trans. Image Process..

[49] Wang K., Peng X., Yang J., Lu S., Qiao Y. Suppressing uncertainties for large-scale facial expression recognition. Proceedings of the IEEE/CVF Conference on Computer Vision and Pattern Recognition.

[50] Vo T.H., Lee G.S., Yang H.J., Kim S.H. (2020). Pyramid with super resolution for In-the-Wild facial expression recognition. IEEE Access.

[51] Zhao Z., Liu Q., Zhou F. Robust lightweight facial expression recognition network with label distribution training. Proceedings of the AAAI Conference on Artificial Intelligence.

[52] Savchenko A.V. (2021). Facial expression and attributes recognition based on multi-task learning of lightweight neural networks. arXiv.

[53] Li H., Sui M., Zhao F., Zha Z., Wu F. (2021). MViT: Mask Vision Transformer for Facial Expression Recognition in the wild. arXiv.

[54] Li Y., Lu Y., Li J., Lu G. Separate loss for basic and compound facial expression recognition in the wild. Proceedings of the Asian Conference on Machine Learning (PMLR).

[55] Chen Y., Wang J., Chen S., Shi Z., Cai J. Facial motion prior networks for facial expression recognition. Proceedings of the 2019 IEEE Visual Communications and Image Processing (VCIP).

[56] Chen S., Wang J., Chen Y., Shi Z., Geng X., Rui Y. Label distribution learning on auxiliary label space graphs for facial expression recognition. Proceedings of the IEEE/CVF Conference on Computer Vision and Pattern Recognition.

[57] Kollias D., Cheng S., Ververas E., Kotsia I., Zafeiriou S. (2020). Deep neural network augmentation: Generating faces for affect analysis. Int. J. Comput. Vis..

[58] Ding H., Zhou P., Chellappa R. Occlusion-adaptive deep network for robust facial expression recognition. Proceedings of the 2020 IEEE International Joint Conference on Biometrics (IJCB).

[59] Cai J., Meng Z., Khan A.S., O’Reilly J., Li Z., Han S., Tong Y. Identity-free facial expression recognition using conditional generative adversarial network. Proceedings of the 2021 IEEE International Conference on Image Processing (ICIP).

[60] Meng Z., Liu P., Cai J., Han S., Tong Y. Identity-aware convolutional neural network for facial expression recognition. Proceedings of the 2017 12th IEEE International Conference on Automatic Face & Gesture Recognition (FG 2017).

[61] Yan K., Zheng W., Zhang T., Zong Y., Tang C., Lu C., Cui Z. (2019). Cross-domain facial expression recognition based on transductive deep transfer learning. IEEE Access.

[62] Aouayeb M., Hamidouche W., Soladie C., Kpalma K., Seguier R. (2021). Learning Vision Transformer with Squeeze and Excitation for Facial Expression Recognition. arXiv.

[63] Wu F., Pang C., Zhang B. (2021). FaceCaps for facial expression recognition. Comput. Animat. Virtual Worlds.

